# The dual distinct role of telomerase in repression of senescence and myofibroblast differentiation

**DOI:** 10.18632/aging.203246

**Published:** 2021-07-12

**Authors:** Masanori Harada, Biao Hu, Jeffrey Lu, Jing Wang, Andrew E. Rinke, Zhe Wu, Tianju Liu, Sem H. Phan

**Affiliations:** 1 Department of Pathology, University of Michigan Medical School, Ann Arbor, MI 48109, USA; 2 Department of Respiratory Medicine, Fujieda Municipal General Hospital, Fujieda, Japan; 3 Xinjiang Key Laboratory of Respiratory Disease Research, Traditional Chinese Medicine Affiliated Hospital of Xinjiang Medical University, Urumqi 830000, China

**Keywords:** cellular senescence, telomerase, myofibroblast

## Abstract

Many aging related diseases such as cancer implicate the myofibroblast in disease progression. Furthermore genesis of the myofibroblast is associated with manifestation of cellular senescence of unclear significance. In this study we investigated the role of a common regulator, namely telomerase reverse transcriptase (TERT), in order to evaluate the potential significance of this association between both processes. We analyzed the effects of TERT overexpression or deficiency on expression of *CDKN2A* and *ACTA2* as indicators of senescence and differentiation, respectively. We assess binding of TERT or YB-1, a repressor of both genes, to their promoters. TERT repressed both CDKN2A and ACTA2 expression, and abolished stress-induced expression of both genes. Conversely, TERT deficiency enhanced their expression. Altering *CDKN2A* expression had no effect on *ACTA2* expression. Both TERT and YB-1 were shown to bind the *CDKN2A* promoter but only YB-1 was shown to bind the ACTA2 promoter. TERT overexpression inhibited *CDKN2A* promoter activity while stimulating YB-1 expression and activation to repress *ACTA2* gene. TERT repressed myofibroblast differentiation and senescence via distinct mechanisms. The latter was associated with TERT binding to the *CDKN2A* promoter, but not to the *ACTA2* promoter, which may require interaction with co-factors such as YB-1.

## INTRODUCTION

The myofibroblast plays important roles in both development and tissue remodeling [1–8]. In the latter case, because of its role as a source of extracellular matrix and pro-fibrotic as well as pro-inflammatory factors, the emergence and fate of the myofibroblast are considered to be of considerable importance in determination of the remodeling outcome [2, 9]. Consistent with differentiated cells [10], myofibroblast differentiation is also accompanied by evidence of cell cycle arrest and even senescence, but is found to be resistant to apoptosis [1, 11–14]. Thus, its persistence in this state can perpetuate the production of cytokines and other factors, which are also constituents of the senescence associated secretory phenotype (SASP) [15]. Interestingly the use of senolytics targeting pro-survival molecules, such as Bcl-2, in myofibroblasts induces apoptosis in these cells [16], consistent with a previous observation that NO-induced apoptosis in these cells is accompanied by reduction in Bcl-2 expression [14]. Recent evidence suggests that treatment with such agents to target the myofibroblast could be of potential benefit in control of aging related diseases characterized by excessive or persistent tissue remodeling [17].

The importance of telomerase in cell differentiation and senescence extends to the myofibroblast. Thus myofibroblast differentiation is associated with decline in telomerase expression, and can in fact be induced or inhibited by TERT deficiency or over-expression, respectively [18–20]. Due to association of the differentiation with expression of senescent marker genes, such as *CDKN2A*, it can be inferred that this manifestation of senescence is also subject to regulation by TERT. Indeed, over-expression of TERT is also associated with reduction in *CDKN2A* expression [21, 22]. These and other studies implicate *CDKN2A* as a potential factor in mediating the cell cycle arrest associated with cell differentiation [23, 24]. However, it is unclear how this regulation is affected and whether the impact on differentiation and senescence are interconnected or mutually interdependent as recently suggested by binding and functional interactions between a myogenic transcription factor and TERT [25, 26].

The significance of telomerase regulation of both myofibroblast differentiation and senescence extends to current understanding of pathogenesis in chronic fibrotic disease wherein the emergence and persistence of the myofibroblast is a key feature. TERT is expressed in stem and progenitor cells in normal tissues but is undetectable in normal adult human somatic cells due to transcriptional repression [27, 28]. However, telomerase activity is induced in certain conditions, including in response to injury, such as in lung fibroblasts from interstitial lung disease patients and animal models of lung injury and fibrosis, but not in myofibroblasts [29]. Thus, telomerase induction is associated with the early proliferative phase after injury, wherein it is associated with the expansion of the fibroblast population. Furthermore, the TERT-deficient mouse lung fibroblasts displayed decreased cell proliferative capacity and higher susceptibility to induced apoptosis compared with control cells [30]. However, during the later stage of myofibroblast differentiation and accompanying senescence marker expression, these cells exhibit diminished telomerase expression. Notably, TERT deficiency induces myofibroblast differentiation, which is repressed by TERT over-expression [18, 22]. Thus these *in vivo* findings of negative association between telomerase vs, myofibroblast differentiation and senescence parallel the *in vitro* findings in cells as noted above. These observations suggest a role for TERT in regulation of cell fate of potential relevance to fibrogenesis. Additionally, certain telomere associated disorders such as aplastic anemia, autosomal dominant dyskeratosis congenita are often associated with fibrotic diseases, including lung and liver fibrosis [31, 32]. Although the precise mechanisms are not fully understood, they may involve the expected short telomere triggering of the DNA damage response with accompanying elicitation of signaling cascades to induce senescence or apoptosis with negative consequences on repair/regeneration responses [32]. Since TERT expression confers a proliferative advantage it has a salutary effect in processes requiring regeneration, such as wound healing [33]. However, the reactivation of TERT expression in many cancers would bypass the checkpoint signaling pathway activated by critical telomere shortening leading to cellular senescence or apoptosis. Cellular senescence can induce detrimental effects in target organs associated with certain diseases, such as idiopathic pulmonary fibrosis (IPF), obesity and type2 diabetes sarcopenia [15]. Many of the chronic fibrotic diseases are prevalent primarily in older adults [34–36], suggesting some association between aging, cell senescence and fibrosis [37]. In the case of IPF, lung fibroblasts exhibit evidence of cellular senescence with higher expression of β-galactosidase, CDKN1A, CDKN2A, p53 and cytokines related to the SASP [15]. In addition to the stress-induced senescence in response to injury, ageing-associated replicative senescence is also a potential contributory factor. The effects of TERT on senescence and differentiation are not fully understood mechanistically despite its catalytic importance in telomere maintenance. In addition to effects related to this canonical telomere maintenance function, TERT exhibits non-canonical functions of potential import in development, cell differentiation and certain disease processes [38–42]. However, it remains unclear how these properties of TERT specifically suppress cell differentiation and senescence, or whether these effects are interdependent.

In this study, we attempted to clarify the significance of senescence in the regulation of myofibroblast differentiation by analyzing how TERT regulates both processes. We examined the effects of altering TERT expression on myofibroblast differentiation and senescence, and analyzed the simultaneous impact of altering *CDKN2A* expression on myofibroblast differentiation to see if differentiation depends on this marker of senescence. Additionally, we assessed the ability of TERT to regulate transcription of *ACTA2* and *CDKN2A* genes as markers of myofibroblast differentiation and senescence, respectively. We also analyzed the role of the transcription factor, Y-box binding protein-1 (YB-1), a known repressor of *ACTA2* gene expression with similar functions to TERT in promotion of cell proliferation through activation of cyclin D1 and repression of cell senescence markers, such as CDKN1A, CDKN2A, p15, p27 and p53 [43–45]. The findings suggested that TERT repressed myofibroblast differentiation and cellular senescence, at least in part by independently repressing *ACTA2* and *CDKN2A* expression, respectively.

## RESULTS

### TERT inhibited myofibroblast differentiation and cellular senescence

**Figure 1:**
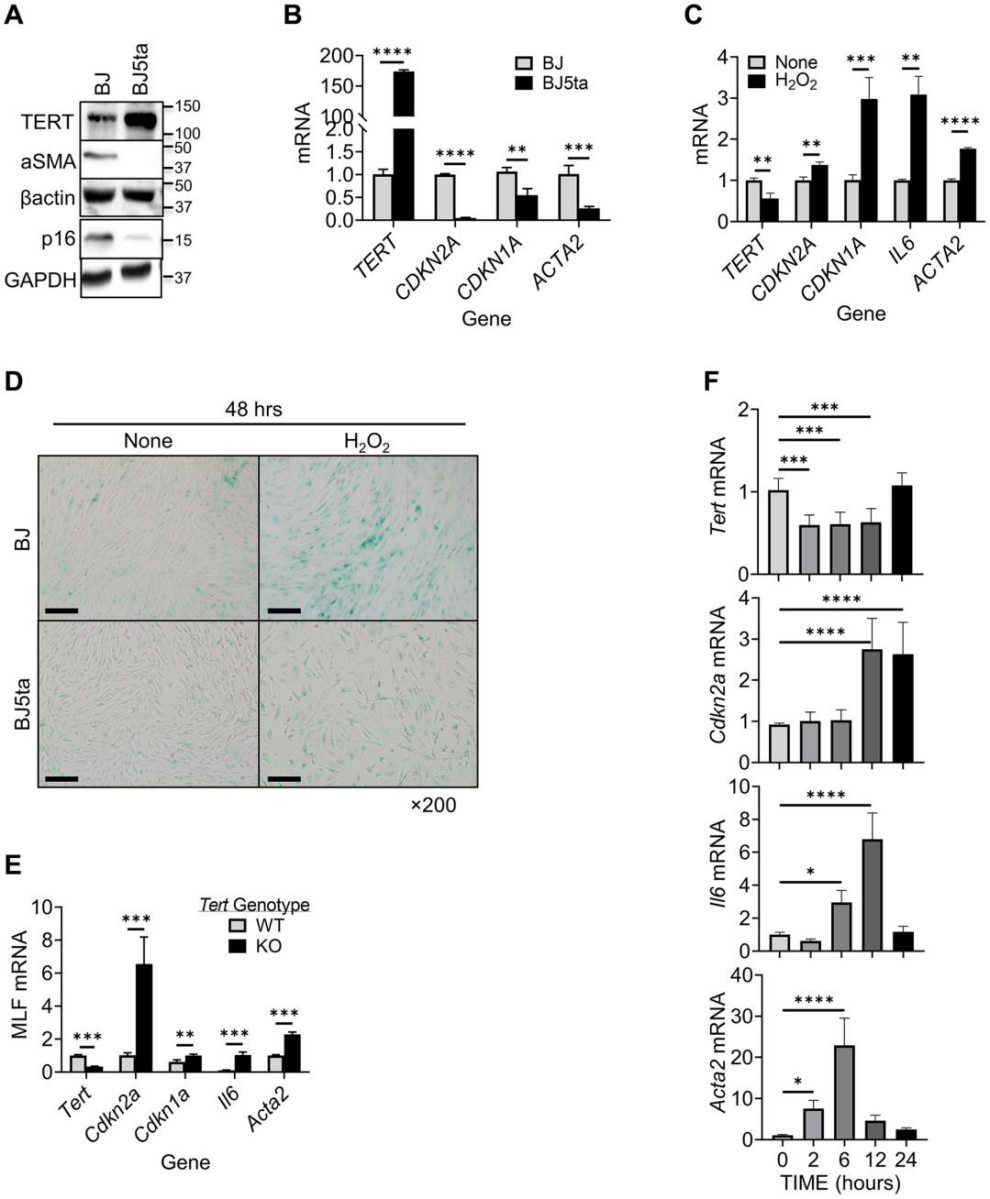
**TERT expression was negatively correlated with αSMA and p16 expression in fibroblasts.** (**A**) Cell lysates were analyzed for TERT, αSMA and p16 proteins by Western blotting. Representative blots from 3 separate experiments are shown. (**B**) RNA samples were analyzed for *TERT*, *CDKN2A,* *CDKN1A* and *ACTA2* mRNAs by qPCR and expressed as 2^−ΔΔCT^. The fold change relative to BJ cells is shown for each gene. (**C**) BJ cell RNA was collected after 6 hours of treatment with H_2_O_2_, and analyzed for *TERT*, *CDKN1A, CDKN2A, IL6,* and *ACTA2* by qPCR. (**D**) Cellular senescence was examined by SA-β-gal staining in cultured cells. A representative image set is shown. Scale bars: 100 μm. (**E**) MLF RNA was analyzed for *Tert*, *Cdkn2a, Cdkn1a, Il6* and *Acta2* by qPCR. (**F**) Primary cultured MLFs were treated with BLM for indicated hours and then analyzed for *Tert*, *Cdkn2a, Il6,* and *Acta2* by qPCR. *N* = 3–4 in (**B**) and (**C**), *N* = 3–5 in (**E**) *N* = 6 in (**F**). ^*^P < 0.05. ^**^P < 0.01. ^***^P < 0.001. ^****^P < 0.0001.

A negative correlation between cellular TERT and αSMA expression is observed in tissue remodeling [46]. We analyzed TERT and αSMA expression levels in human foreskin fibroblasts (BJ) and BJ5ta cells, in which TERT is stably overexpressed in BJ cells. TERT expression was detected at low levels in BJ cells, and highly expressed in BJ5ta cells (Figure 1A). In contrast, αSMA expression was clearly detected in BJ but undetectable in BJ5ta cells. Notably the level of p16 expression parallels that for αSMA in these 2 cell lines. This negative correlation was also observed at the mRNA level when comparing BJ vs. BJ5ta cells. Similarly to p16 (*CDKN2A*), another senescence marker p21 (*CDKN1A*) mRNA also showed negative correlation with αSMA (*ACTA2*) (Figure 1B).

We examined next the effects of hydrogen peroxide (H_2_O_2_) induced cellular senescence, and possible concomitant myofibroblast differentiation [47]. The results showed suppression of TERT expression by H_2_O_2_ in BJ cells (Figure 1C). This was in contrast to the stimulatory effect of H_2_O_2_ on both p16/p21 and αSMA expression, accompanied by increase in SASP marker IL6 mRNA. In TERT overexpressed BJ5ta cells, H_2_O_2_ did not have a significant effect on expression of any of these genes (not shown). H_2_O_2_ also induced high level of senescence associated (SA) β-gal activity in BJ, which was barely detectable in BJ5ta cells (Figure 1D). These results suggested that TERT might be a repressor of both *ACTA2* and *CDKN2A* expression. The effects of TERT deficiency on p16, p21, IL6 and αSMA expression were then examined in MLF from wild type vs. TERT deficient mice. Cells from TERT floxed mice exhibited 70% reduction in TERT expression when transduced with Cre-bearing adenovirus (Figure 1E). These TERT-deficient MLF exhibited >6-fold increase in p16, >1.6-fold in p21, >10-fold in IL6, and >2-fold increase in αSMA expression, which would be consistent with TERT as a repressor of these genes.

To further examine if this response was unique to oxidative stress-induced senescence, we analyzed the effects of bleomycin (BLM), an alternate inducer of senescence. Similar to H_2_O_2_ -induced cellular senescence, BLM caused prompt (at 2 hours) significant reduction in TERT mRNA, but delayed increase in p16 mRNA at 12 and 24 hours, and IL6 mRNA at 6 and 12 hours, respectively (Figure 1F). αSMA mRNA was also significantly increased by BLM, but more rapidly (≥2 hours) than p16. These results suggested TERT inhibited myofibroblast differentiation and senescence albeit with different kinetics.

**Figure 2:**
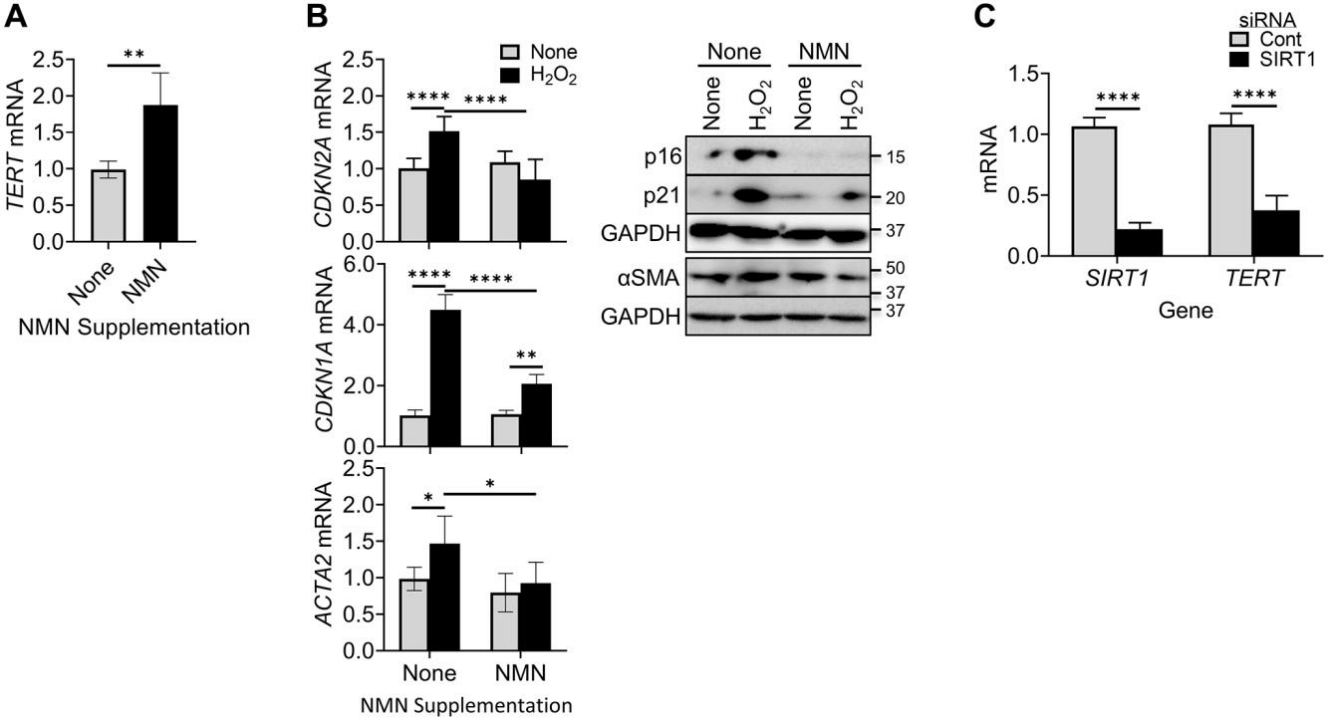
**NMN increased TERT expression but reduced cellular senescence and myofibroblast differentiation.** (**A**) BJ cells were treated with NMN, and then analyzed for *TERT* by qPCR. (**B**) BJ cells were treated with NMN followed by H_2_O_2_ induction, Cells collected at 6 hours or 48 hours after H_2_O_2_ treatment, were analyzed for *CDKN2A* and *ACTA2* mRNAs by qPCR (left) or their respective protein expressions by western blotting (right). (**C**) Lung fibroblasts were transfected with 20 nM *SIRT1* or control siRNA, and were analyzed for *TERT* and *SIRT1* mRNA by qPCR. *N* = 5 in (**A**), in (**B**) and 4 in (**C**). ^*^P < 0.05. ^**^P < 0.01. ^****^P < 0.0001.

We next explore the potential impact of NAD^+^ on TERT regulation of senescence by evaluating the effects of increasing intracellular NAD^+^ levels using NMN supplementation. NAD^+^ is a co-substrate for Sirt1, an inducer of TERT [48–50]. The results showed that TERT expression was significantly enhanced by NMN treatment (Figure 2A). This increased TERT expression by elevating cellular NAD^+^ was associated with reduced senescence and differentiation responses as manifested by the lack of increase in p16, p21, and αSMA expression, respectively, at both mRNA and protein levels (Figure 2B). Interestingly knockdown (~80%) of SIRT1 expression using SIRT1 siRNA caused a significant >70% reduction in TERT mRNA (Figure 2C). These findings suggested that increase of TERT suppressed cellular senescence and myofibroblast differentiation, likely mediated by increasing SIRT1 activity.

### TERT repressed αSMA expression in a p16 independent manner

**Figure 3:**
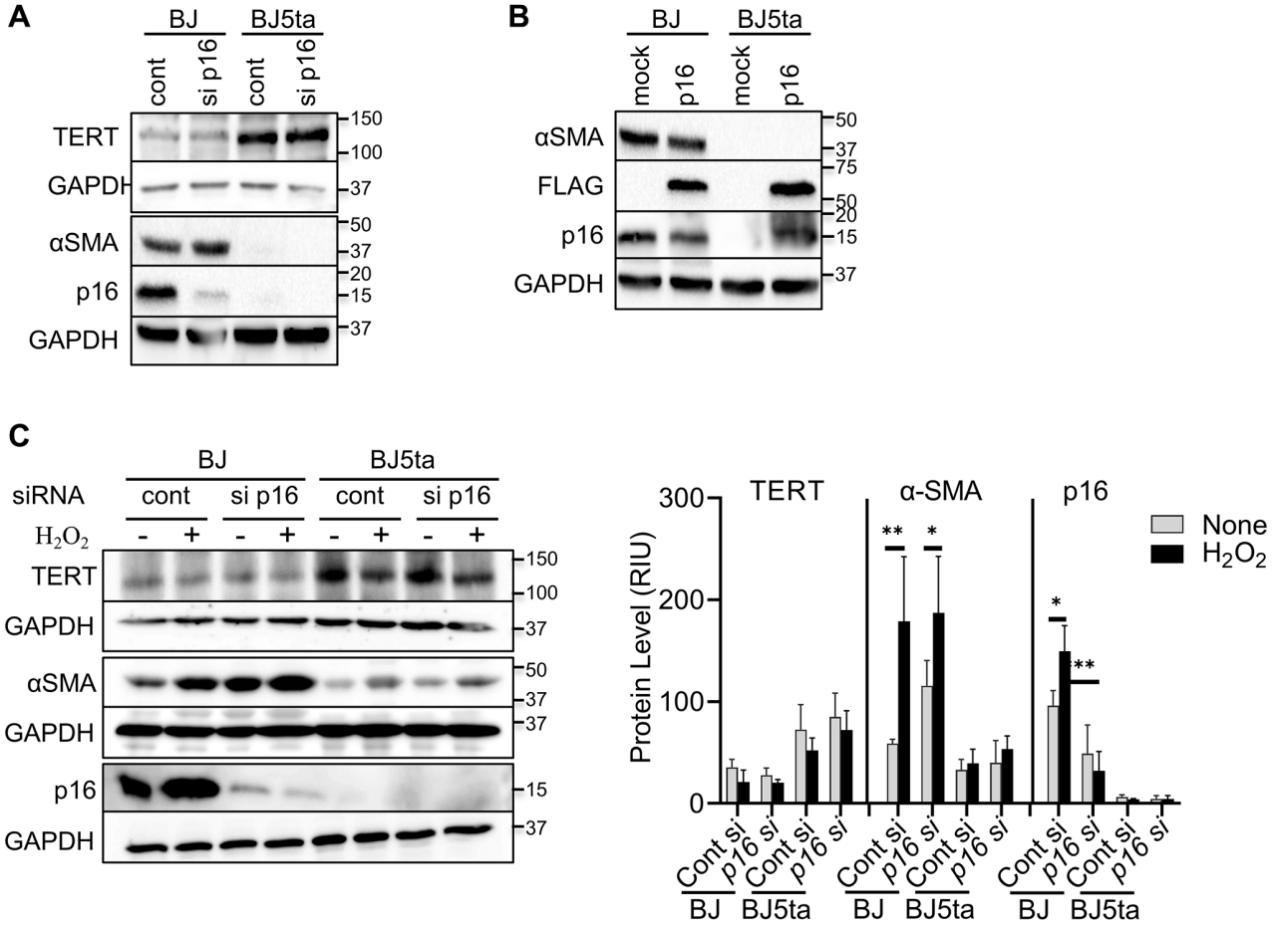
***TERT* repressed αSMA expression in a p16 independent manner.** (**A**) Control or p16 siRNA transfected cells were analyzed for the indicated proteins by western blotting. (**B**) Cells were transfected with pCMV-Myc-DDK-*CDKN2A* plasmid or control vector and analyzed as in (**A**). DDK (FLAG^®^) antibody was used to confirm the level of transfection. (**C**) The cells were transfected with p16 siRNA, followed by with H_2_O_2_ treatment for 48 hours, and then analyzed as in (**A**). The cell lysates were analyzed for αSMA and p16 protein by western blotting. GAPDH were used as the internal controls for all blots. The quantified protein expression level was expressed as relative integration units (RIU). Representative blots from 3 separate experiments are shown. ^*^P < 0.05. ^**^P < 0.01.

To determine whether TERT repression of αSMA expression was dependent on repression of p16, we analyzed the effects of p16 knockdown or over-expression on αSMA protein expression. Consistent with the findings in Figure 1, there was negative correlation between TERT and both αSMA and p16 expression, which was not significantly affected by transfection of p16 siRNA (Figure 3A). However, notably p16 knockdown did not have an effect on αSMA expression in BJ cells despite the reduction in p16 expression. Both p16 and αSMA expression was barely detectable in BJ5ta cells with or without p16 siRNA treatment. As with the knockdown experiment, p16 overexpression also failed to affect TERT or αSMA expression (Figure 3B). The repression of αSMA expression by TERT in the BJ5ta cells could not be overcome by p16 overexpression, which was readily detectable in the BJ5ta cells transfected with the p16 expression plasmid. These results suggested that TERT repression of αSMA expression was independent of its effect on p16 expression. Next, we analyzed the effects of p16 siRNA on αSMA expression in H_2_O_2_-treated cells. H_2_O_2_ caused >40% reduction in TERT expression, although it did not reach statistical significance. αSMA and p16 expression was significantly increased by H_2_O_2_ treatment only in the BJ cells (Figure 3C). Knockdown of p16 did not have a significant effect on αSMA expression in control or H_2_O_2_ treated cells. Thus premature cellular senescence induced αSMA expression was repressed by TERT but was independent of p16.

### TERT binding to *CDKN2A but not to ACTA2* gene promoter

**Figure 4:**
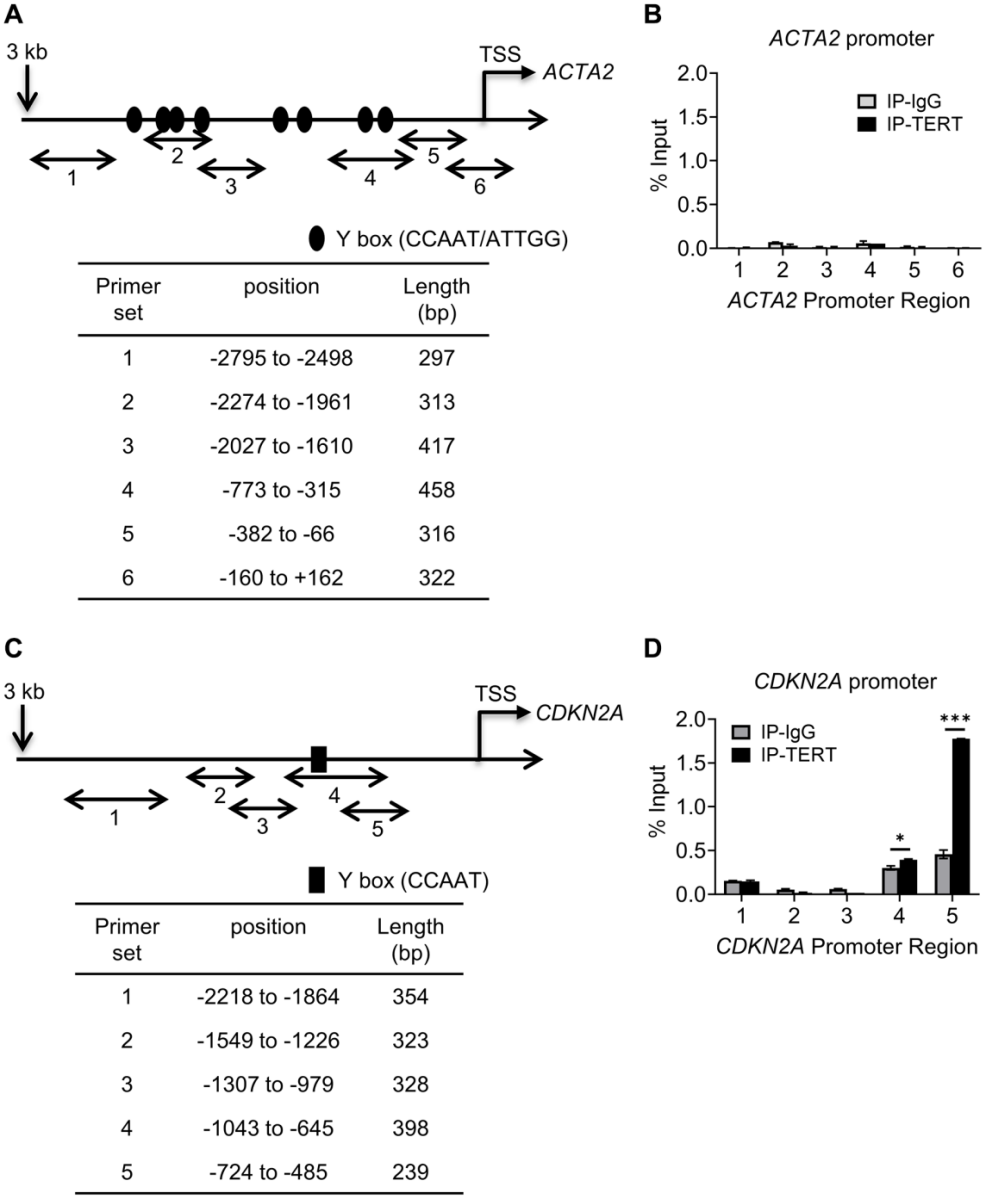
**TERT binding to *CDKN2A* but not to *ACTA2* promoter.** Schematic illustration of *ACTA2* (**A**) and *CDKN2A* (**C**) promoters are shown. PCR primers for ChIP assays are indicated by numbered bidirectional arrows, with their positions and lengths listed in the respective tables below the illustrations in (**B**) and (**D**). ChIP assays to assess TERT binding to *ACTA2* (**B**) or *CDKN2A* (**D**) promoter in BJ5ta cells were performed using primers as numbered in (**A**) and (**C**), respectively. The cell DNA immunoprecipitated by YB-1 antibody was amplified by qPCR. One tenth of the supernatant before immunoprecipitation was used for the DNA input control. Results are expressed as % of input. *N* = 3 each group. ^*^P < 0.05. ^***^P < 0.001.

We next determined if repression involved direct binding of TERT to either of their gene promoters. We checked for binding to the promoter regions upstream from the respective transcription start sites of the *ACTA2* and *CDKN2A* genes, using 5-6 randomly constructed partially overlapping primer sets (Figure 4A and 4C). The ChIP assays using anti-TERT antibodies failed to detect any binding of TERT to the *ACTA2* promoter, but did show binding to regions covered by primer sets 4 and 5 of the *CDKN2A* promoter (Figure 4B and 4D). Most of the binding to the *CDKN2A* promoter occurred at the more proximal site 5. These results suggested that TERT was unlikely to directly regulate *ACTA2*, but could directly repress the *CDKN2A* gene.

### Role of YB-1 in TERT repression of *ACTA2* and *CDKN2A* gene expression

**Figure 5:**
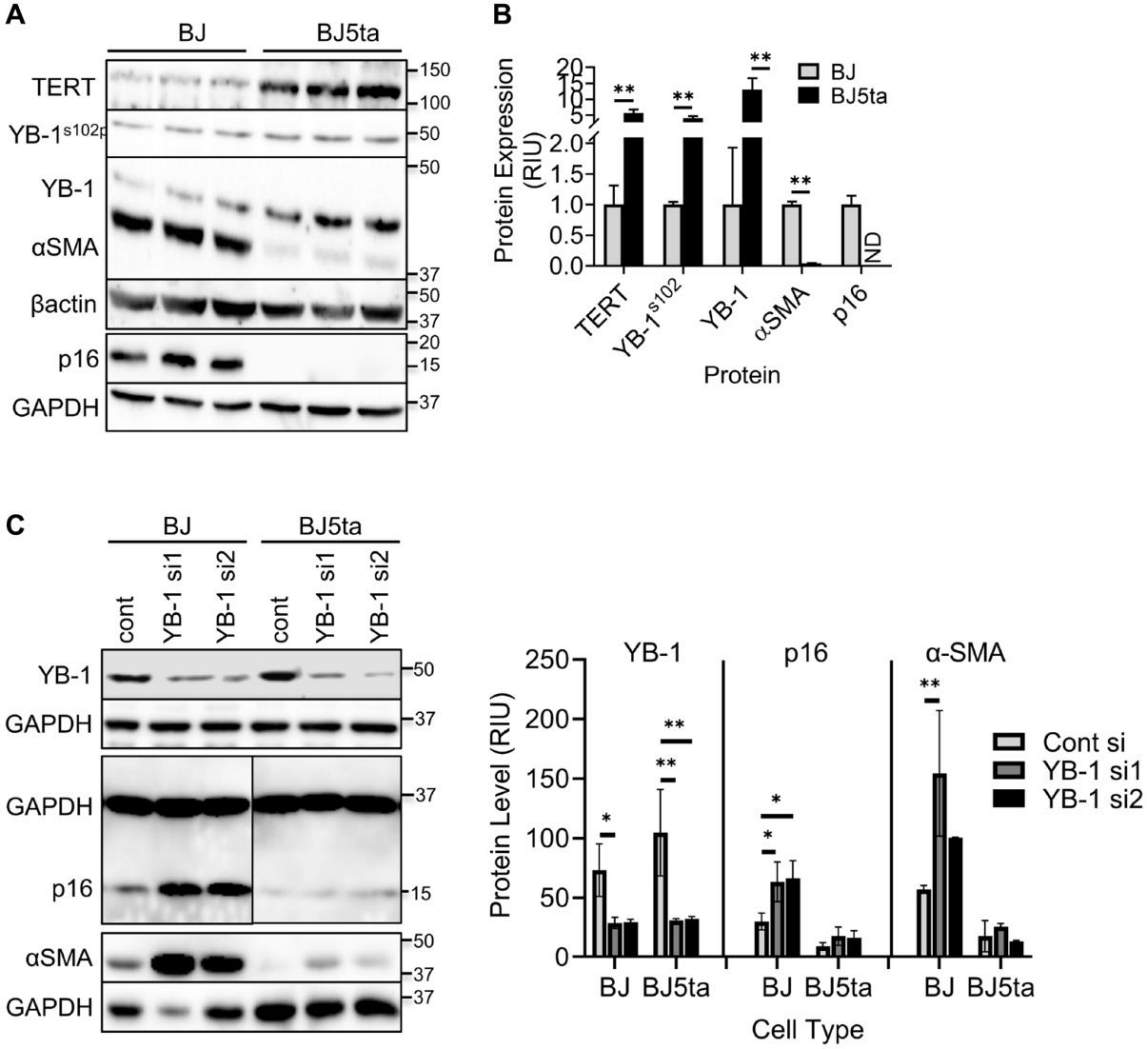
**TERT enhanced YB-1 expression and phosphorylation was associated with repression of *ACTA2* gene expression.** (**A**) Expression of TERT, αSMA, p16, YB-1 and phosphorylated YB-1 (YB-1^S102P^) in whole cell lysates was analyzed by western blotting and the results of quantitative analysis shown as relative integration units (RIU) (**B**). β-actin or GAPDH was used as the internal control. ND, not detected. *N* = 3 each group in (**B**). (**C**) Control or YB-1 siRNA transfected BJ or BJ5ta cells were analyzed for expression of indicated proteins by western blotting, and the corresponding quantified protein levels are shown in the right panel. Representative blots from 3 separate experiments are shown. ^*^P < 0.05. ^**^P < 0.01.

The lack of detectable binding interaction between TERT and the *ACTA2* promoter suggests possible mediation by interaction with another transcription factor. Analysis of the promoter sequences of both *ACTA2* and *CDKN2A* genes revealed presence of multiple Y-box sequences (Figure 4A and 4C). The results showed that in contrast to reduced αSMA and p16 expression, the overexpression of TERT in BJ5ta cells was associated with increased Y-box binding protein, phosphorylated YB-1^S102P^ and YB-1 expression relative to that in BJ cells (Figure 5A and 5B), while repressing both αSMA and p16 protein expression. Next the effects of YB-1 knockdown using siRNA was examined. In BJ cells YB-1 deficiency caused substantial increase in expression of αSMA and p16, but with only minor effects in BJ5ta cells (Figure 5C). Thus the TERT-associated repression of both αSMA and p16 expression in the BJ5ta cells could not be fully overcome by the knockdown of YB-1, suggesting distinct mechanisms of repression of ACTA2 by TERT and YB-1, with repression by TERT being more potent than that exerted by YB-1.

### YB-1 binding to *ACTA2* and *CDKN2A* promoters

**Figure 6:**
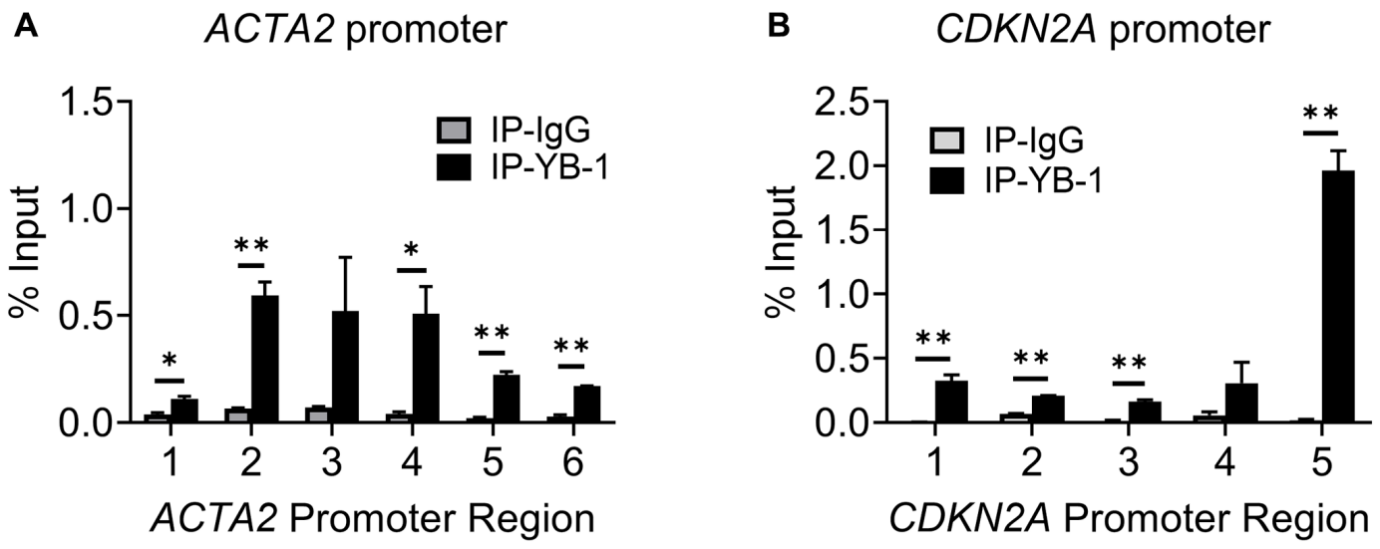
**YB-1 directly binds to *ACTA2* and *CDKN2A* promoters.** ChIP assays to assess YB-1 binding to *ACTA2* (**A**) or *CDKN2A* (**B**) promoter in BJ5ta cells were undertaken using the same primers in 4A and C, respectively. The cell DNA immuneprecipitated by YB-1 antibody was amplified by qPCR. One tenth of the supernatant before immunoprecipitation was used for the DNA input control. Results are expressed as % of input. *N* = 3 each group. ^*^P < 0.05. ^**^P < 0.01.

Multiple putative YB-1 binding sites (Figure 4A and 4C) could be identified in the upstream regulatory regions of both *ACTA2* and *CDKN2A* genes, respectively. While 8 Y-box were identified in the *ACTA2* promoter, only a single Y-box motif was present in the *CDKN2A* promoter. We used the same primer sets in Figure 4A and 4C to undertake ChIP assays using anti-YB-1 antibodies. YB-1 binding was detected primarily at the regions in the *ACTA2* promoter covered by primer sets 2-4 (Figure 6A). Binding to the *CDKN2A* gene promoter was also observed with the strongest signal in the most proximal region covered by primer set 5, which notably did not contain a Y-box sequence (Figure 6B), but where TERT was also shown to bind (Figure 4D). These results suggested that YB-1 repression of *ACTA2* expression was associated with direct binding to its promoter. However in the case of repression of the *CDKN2A* gene, both YB-1 and TERT could bind its promoter but at distinct sites except for the region covered by primer set 5.

**Figure 7:**
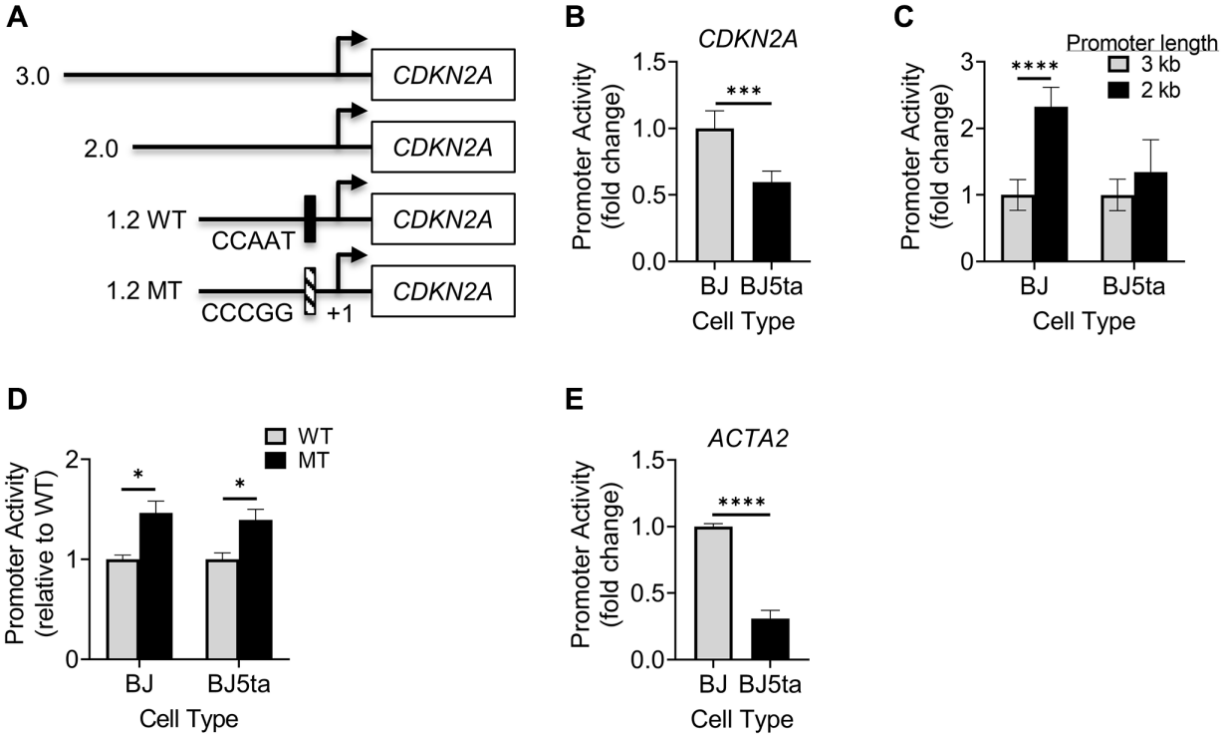
**Analysis of *CDKN2A* or *ACTA2* promoter activity.** (**A**) Schematic illustration shows 4 promoter constructs with different lengths and location of Y-box mutation. The indicated *CDKN2A* promoter constructs were transfected into cells, and the promoter-luciferase activity was measured for the 3 kb construct in BJ vs. BJ5ta cells (**B**), 3 and 2 kb promoters (**C**), and 1.2 kb WT vs. MT in both cells (**D**). (**E**) The *ACTA2* promoter construct was measured similarly as (**B**). All values shown were after normalizing to the *Renilla* luciferase activity. The results were expressed as relative light units. *N* = 5 in (**B**), 6 in (**C** and **D**), and 3 in (**E**). ^*^P < 0.05. ^***^P < 0.001. ^****^P < 0.0001.

To further investigate the transcriptional regulatory mechanisms, we compared the activity of 4 *CDKN2A* promoter constructs. The 3 wild type constructs of varying lengths, namely 3, 2 and 1.2 kb, plus a Y-box mutated version of the 1.2 kb promoter, are illustrated in Figure 7A. Compared to that in BJ cells, the 3 kb construct exhibited significantly lower activity in BJ5ta cells (Figure 7B), confirming TERT regulation of p16 at the transcriptional level. The 2 kb construct with truncation of the most distal 1 kb resulted in significant increase in promoter activity in BJ, but not in BJ5ta cells (Figure 7C). In contrast, the 1.2 kb promoter activity was significantly increased upon mutation of the Y-box in both cells (Figure 7D), suggesting a significant role for YB-1 binding in repression of *CDKN2A* transcription even in the presence of TERT over-expression.

Finally, analysis of the activity of the ACTA2 promoter in BJ vs. BJ5ta cells showed that ACTA2 promoter activity was significantly decreased by ~70% in BJ5ta cells compared to BJ cells (Figure 7E), suggesting that TERT repressed ACTA2 at the transcriptional level.

## DISCUSSION

**Figure 8:**
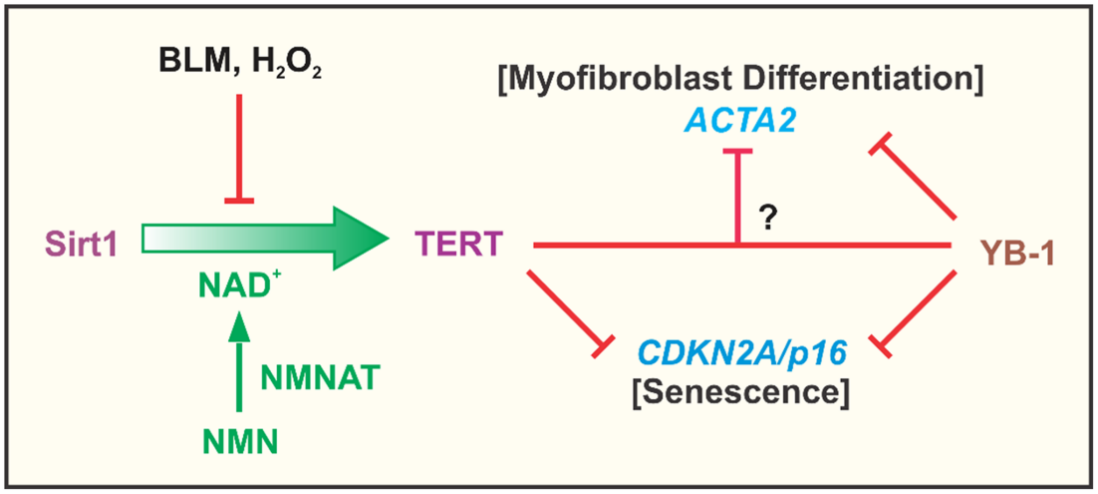
**Summary cartoon.** The findings from the study confirmed the importance of NAD^+^ dependent Sirt1 in regulation of TERT expression in fibroblasts, which was suppressed by the cellular senescence inducing agents, BLM and H_2_O_2_. This suppression was due apparently to depletion of NAD^+^ since it was reversed by NMN supplementation to increase intracellular NAD^+^ upon conversion by nicotinamide nucleotide adenylyltransferase (NMNAT). Elevated TERT expression such as in BJ5ta fibroblasts, potently inhibited *ACTA2* and *CDKN2A* gene expression, with evidence of its direct binding to the *CDKN2A* but not the *ACTA2* promoter. TERT inhibition of ACTA2 expression, did not depend on CDKN2A, suggesting that TERT regulation of myofibroblast differentiation was independent of its effects on senescence. YB-1, a known suppressor of both CDKN2A and ACTA2 expression, exhibited binding interactions to the promoters of both genes. Additionally or alternatively, it might interact with TERT to inhibit ACTA2, however inhibition by TERT could not be overcome by depletion of YB-1.

Telomeres and telomerase are key determinants of aging and aging-related diseases [51, 52]. Telomerase/TERT is activated in many cancerous cells, and implicated in regulation of cell proliferation, fate and differentiation, which extends to fibroblasts and myofibroblast differentiation [19, 30, 40–42, 53]. However the underlying mechanism and its relationship to cellular senescence remain unclear. In this paper we analyzed the role of TERT in regulating the expression of *ACTA2* and *CDKN2A* genes as indicators of myofibroblast differentiation and cellular senescence, respectively. The findings suggested that TERT expression was negatively correlated with both myofibroblast differentiation and senescence. This would be consistent with the role of TERT in cell proliferation and inhibition of cell senescence. TERT upregulation by NMN was diminished by Sirt1 knockdown indicating that the NMN effect was mediated via enhancement of Sirt1 activity. In contrast TERT deficiency in fibroblasts reduced fibroproliferation and tissue remodeling of potential import in cancer [30]. The dual role of repressing myofibroblast differentiation while promoting fibroblast proliferation could account for the known association between differentiation and cell cycle arrest. These were summarized in Figure 8. Notably, TERT expression or its re-induction in the case of iPSC generation, is a marker of ‘stemness’ of potential relevance for maintenance of cancer stem cells [54, 55]. The role of fibroblast activation/myofibroblast differentiation in cancer is not clear. The heterogeneity of cancer-associated fibroblasts (CAFs) in pancreatic ductal adenocarcinoma may play different roles in a study using an organoid culture system. While periglandular αSMA^high^ myofibroblastic CAFs likely restrain tumor growth, diffusely distributed αSMA^low^ IL-6–positive inflammatory CAFs promote tumor growth possibly by secreting ECM and cytokines such as IL-6 [56, 57]. Interestingly, TERT is expressed primarily in αSMA^low^ less differentiated or undifferentiated fibroblasts in injured lung tissue [29]. Thus, elucidation of TERT regulation of myofibroblast differentiation and senescence may provide insight into the basis for the difficulty in targeting fibroblasts in cancer therapy. Inhibition of fibroblast telomerase activity is sufficient to induce myofibroblast differentiation [19], while TERT deficiency reduces cell proliferative capacity [30]. Moreover, stimulation of myofibroblast differentiation by TGFβ causes Smad3 dependent repression of TERT expression [58]. This significant impact of TERT on cell differentiation, proliferation and fate suggests potential mechanisms by which manipulation or control of Sirt1 activity, such as via NMN supplementation, could impact on aging and senescence, as well as aging-associated chronic diseases. The dual effect of TERT on differentiation and senescence also signify that processes (e.g. tissue repair/remodeling) that require genesis of abundant myofibroblasts would require initial stimulation of progenitor cell or fibroblast proliferation associated with high TERT expression, followed by subsequent differentiation with cell cycle arrest under low TERT conditions. It remains unclear if these are interdependent effects accounting for the noted cell cycle arrest associated with cell differentiation. The findings from our study indicated that TERT inhibition of myofibroblast differentiation did not depend on inhibition of senescence. Moreover, over-expression of p16 failed to overcome TERT inhibition of αSMA expression.

The noted negative correlation between TERT expression vs. both αSMA and p16 expression suggested repression of gene transcription. However direct binding of TERT to the promoters could only be demonstrated with the *CDKN2A* promoter, suggesting potential direct repression of *CDKN2A* but not *ACTA2* transcription. However, *ACTA2* promoter activity was clearly repressed by TERT over-expression consistent with transcriptional repression. Analysis of these two promoters identified putative Y-boxes, which could bind YB-1, a known repressor of both αSMA and p16 expression. Indeed YB-1 was shown to bind to multiple sites on the 2 promoters. Interestingly the predominant binding to the *CDKN2A* promoter coincided with the location of primary binding site for TERT, suggesting potential binding interaction between the two proteins in mediating repression of transcription. The sole Y-box in the *CDKN2A* promoter was functionally important since its mutation results in increased promoter activity, indicating that YB-1 repressed transcription was dependent at least in part by binding to this site. Given the binding to the *ACTA2* promoter, YB-1 could potentially also recruit TERT to interact in combinatorial fashion and cooperatively repress transcription. This is supported by previous evidence of YB-1^s102^ phosphorylation by RSK2 with formation of a RSK2/hTERT/YB-1 complex leading to repression of *CDKN2A* and promotion of soft agar colony formation [59]. An alternative and/or additional mechanism is the enhanced YB-1 expression and activation by TERT over-expression to mediate the indirect TERT repression of αSMA expression. However YB-1 knockdown failed to overcome repression of αSMA or p16 expression by TERT over-expression in BJ5ta cells. Thus TERT repressed αSMA and p16 expression was not dependent on YB-1, suggesting distinct mechanisms were involved with the repression by TERT predominating and being considerably more potent. This would be consistent with the suggestion of differential binding between these two factors on the 2 promoters by constructing complex with other transcriptional factor YB-1 (Figure 4). In addition to inhibition of senescence, YB-1 can also directly bind to the cyclin D1 promoter to promote cell cycle progression [43]. TERT’s effect on YB-1 expression and activation would enhance such an effect on proliferation. Potential interaction with YB-1 to mediate TERT regulation to repress the αSMA expression cannot be ruled out at this time. However, the totality of the findings suggest that TERT repression of both myofibroblast differentiation and cellular senescence did not depend on YB-1, or at least in a measurable way. This would suggest that separate target mechanisms were involved, but with the TERT repressive effect being predominant. Further studies are necessary to precisely map out the targeting of the promoters of both *CDKN2A* and *ACTA2* genes by TERT to mediate repression of transcription.

## MATERIALS AND METHODS

### Cell culture and antibodies

Human foreskin fibroblast cell lines BJ, BJ5ta (ATCC, Manassas, VA, USA) and primary human lung fibroblasts (kind gifts from Dr. Craig A. Henke, University of Minnesota, Minneapolis, MN) were grown in Dulbecco’s modified Eagle’s medium (DMEM) supplemented with 10% fetal bovine serum at 37°C in an atmosphere containing 5% CO_2_. Mouse lung fibroblasts (MLF) were isolated with a digestion cocktail containing collagenase III and DNase I (Worthington Biochemical Crop., Lakewood, NJ, USA), and maintained in DMEM supplemented with 10% plasma-derived fetal bovine serum (PDS; Animal Technologies, Tyler, TX, USA), 10 ng/ml of EGF and 5 ng/ml of PDGF (R & D systems, Inc. Minneapolis, MN, USA) as before [60].

### DNA plasmids and siRNA transfections

BJ and BJ5ta cells were cultured 24 hours prior to transfection. To induce p16 over-expression, the cells were transfected with human *CDKN2A* plasmid with the DDK tag (pCMV- mycDDK- *CDKN2A* plasmid, OriGENE, Rockville, MD) or control plasmid (pCMV) using lipofectamine 3000 (Invitrogen, Carlsbad, CA, USA) at a ratio of 2:3 (μg:μl), and incubated for 48 h at 37°C. The cells were harvested for RNA or protein extraction and analysis as described below.

To induce deficiency of p16, YB-1 or SIRT1, their respective siRNAs (p16, YB-1 from Sigma, pre-designed Silencer^®^ select SIRT1 (Ambion s23771) from Thermo Fisher Scientific) or control siRNA, were used to transfect the fibroblasts using Lipofectamine™ RNAiMAX (Invitrogen), according to the manufacturer’s protocol. The nucleotide sequences were shown in Supplementary Table 1.

### Western blot and qRT-PCR analysis

The antibodies used in this study were as follows: anti- TERT (MBL, Nagoya, Japan), anti-p16, anti-YB-1 (Abcam, Cambridge, MA, USA), YB-1^S102p^ (Cell signaling Technology, Beverly, Massachusetts, USA), anti-p21-HRP (Santa Cruz, CA, USA), anti-αSMA, anti-β-actin and anti-GAPDH (Sigma, St. Louis). Proteins were visualized using an enhanced chemiluminescence system (Perkin Elmer, Waltham, MA, USA) and scanned for quantitative analysis using an Imager 600 with ImageQuant TL software (GE Healthcare, Chicago, IL, USA).

Taqman primers (Thermo Fisher Scientific) were used for qPCR analysis of human and mouse TERT, αSMA, YB-1, p16, p21, IL6, SIRT1 and 18s rRNA. One-step RT-PCR was performed as before [60] using a GeneAmp 7500 sequence detection system (Applied Biosystems, Rockford, IL). Results were expressed as 2^−ΔΔCT^ using the indicated control group as calibrator and 18srRNA as reference [61].

### Induction of cellular senescence

BJ/BJ5ta cells, or MLF were cultured until ≥80% confluent in 10 cm culture dishes. The cells were treated with 200μM H_2_O_2_ (Sigma, St Louis MO, USA) for 2 hours, followed by media removal, washing twice with DMEM and addition of fresh media. After 48 hours the cells were harvested for mRNA or protein analysis. Where indicated some of the cells were treated with 1 mM nicotinamide mononucleotide (NMN) for 48 hours prior to H_2_O_2_ treatment. For BLM induction of senescence, cultured MLF were treated with 50μg/ml BLM for the indicated time-points.

### Promoter activity assay

The human *ACTA2* promoter-luciferase construct (pGL4-*ACTA2*) from -1698 to 89 relative to the transcriptional start sites (TSS) is described as before [62]. Three pGL4-*CDKN2A* promoter-luciferase constructs of different lengths (3 kb, 2 kb, and 1.2 kb upstream from the transcription start site) plus the 1.2 kb version with a Y-box mutation (MT)) were kindly provided by Dr. Yojiro Kotake (Hamamatsu University School of Medicine, Shizuoka, Japan). Each construct (0.5 μg) was co-transfected with 25 ng *Renilla* luciferase control vector (pRL-SV40, Promega Corporation, Madison, WI, USA) into BJ or BJ5ta cells plated in 24-well plates, using Lipofectamine 3000 (Invitrogen). The cells were harvested 48 hours after transfection, and the activity of firefly or *Renilla* luciferase was measured using the dual luciferase reporter assay system (Promega) with a Veritas Microplate Luminometer (Turner Biosystems, Sunnyvale, CA, USA). The relative luciferase activity was calculated by normalizing firefly luciferase activity to that of *Renilla* luciferase. Experiments with each construct were repeated four times, and relative light units were expressed as means ± SD.

### Chromatin immunoprecipitation (ChIP) assay

This was undertaken as before [62] to analyze for TERT binding to the DNA sequence of interest, Briefly, BJ5ta cells (3.0 × 10^6^) were treated with 1% formaldehyde for DNA-protein crosslinking. This was terminated by addition of 0.125 M glycine. The cells were lysed sequentially with cell lysis buffer (50 mM Hepes, pH 7.5, 0.5% NP-40, 140 mM NaCl, 1 mM EDTA, 10% glycerol, 0.25% Triton X-100, and a protease inhibitor cocktail) and after centrifugation with nuclear lysis buffer (10 mM Tris, pH 8.0, 200 mM NaCl, 1 mM EDTA, 0.5 mM EGTA, and a protease inhibitor cocktail). This was followed by sonication and after centrifugation, the lysates were incubated with anti-TERT antibodies (MBL, Nagoya, Japan, clone#, 10E9-2) or control rabbit IgG, followed by addition of Dybabeads^®^ protein G (Thermo Fisher). After reversing the crosslinking, the purified DNA fragments were subjected to PCR using SYBR Select Master Mix (Applied Biosystems) and the products were analyzed by electrophoresis. Primer pairs were shown in Supplementary Tables 2 and 3.

### Statistical analysis

All data were expressed as mean ± SD. Differences between means of various treatment and control groups were assessed for statistical significance by Student’s *t*-test for two group comparisons, or ANOVA with post hoc Tukey test for multiple comparisons using GraphPad Prism (version 8.4.0, GraphPad Software, San Diego, CA, USA) *p* < 0.05 was considered to indicate statistical significance.

### Study approval

All mice were housed in the University Laboratory Animal Facility in accordance with animal protocols approved by the Institutional Animal Care & Use Committee at the University of Michigan.

## Supplementary Materials

Supplementary Tables
