# Supplementary material for: The dual distinct role of telomerase in repression of senescence and myofibroblast differentiation
Source: Aging (Albany NY). 2021 Jul 12;13(13):16957–73. doi: 10.18632/aging.203246 (PMC8312426; doi:10.18632/aging.203246)
Supplement: Supplementary Tables [file aging-13-203246-s001.pdf]

SUPPLEMENTARY TABLES

Supplementary Table 1. Sequences of oligonucleotides used for siRNA transfection.

siRNA	Primers #		Sequences
YB-1	#1	Sense	5′-UGACACCAAGGAAGAUGUAUU
		Antisense	5′-UACAUCUUCCUUGGUGUCAUU
	#2	Sense	5′-GUGAGAGUGGGGAAAAGAAUU
		Antisense	5′-UUCUUUUCCCCACUCUCACUU
CDKN2A		Sense	5′-CGCACCGAAUAGUUACGGUT-3′
		Antisense	5′-ACCGUAUUCGGUGGUGCGTT-3′
SIRT1		Sense	5′-GGGUCUUCCCUCAAAGUAAtt-3′
		Antisense	5′-UUACUUUGAGGGAAGACCCaa-3′

Supplementary Table 2. Primer sets for ChIP assay (*ACTA2*).

ChIP *ACTA2*	Primer sets	Sequences
1	Forward	TTCCTTTGAAACACTGACACTTC
	Reverse	AGACATTAGCAACCAGGATGG
2	Forward	TTCTTTATTTTCCCCACAAAAC
	Reverse	GAGCAGCCTGTTTTGGAAAG
3	Forward	GATCCACATCCCTTCAATTAGC
	Reverse	AATGGGCTGGACATAGATGG
4	Forward	CAGATGCAATCAGCGAACAG
	Reverse	ACTCCCATGGTGATTTCTGC
5	Forward	GTTCTGTGCGGTGGGAAC
	Reverse	GCACCGAAGCAGTGGTTAAG
6	Forward	GCCTCCAGAAGCTCATTCAG
	Reverse	GACAGGAATTGAAGCGGAAG

Supplementary Table 3. Primer sets for ChIP assay (*CDKN2A*).

ChIP CDKN2A	Primer sets	Sequencing
1	Forward	ACTGGGTCTACAAGGTTTAAGTC
	Reverse	CTGGGAGACAAGAGCGAAAC
2	Forward	TCGGAGTCTCATTCTGTCACC
	Reverse	TGGCTTGCAATCTTGTTTTG
3	Forward	GTGCTGGGATTACAGGTGTG
	Reverse	TGCACAGTGATCCAAAGACC
4	Forward	GAGAAATGTGAGAAGTGTGAAGG
	Reverse	CCCCTCAACCCTTGATTTTC
5	Forward	CCGACTCTCCAAAAGGAATC
	Reverse	GTTTCCTTCCTCCGCGATAC
